# Multi-Etiological Nature of Tuberculosis-Like Lesions in Condemned Pigs at the Slaughterhouse

**DOI:** 10.1371/journal.pone.0139130

**Published:** 2015-09-29

**Authors:** Fernando Cardoso-Toset, Jaime Gómez-Laguna, Shyrley P. Amarilla, Ana I. Vela, Librado Carrasco, Jose F. Fernández-Garayzábal, Rafael J. Astorga, Inmaculada Luque

**Affiliations:** 1 Department of Animal Health, Faculty of Veterinary Medicine, ‘International Excellence Agrifood Campus, CeiA3’, Córdoba, Spain; 2 Department of R&D, CICAP - Food Research Centre, Pozoblanco, Córdoba, Spain; 3 Department of Anatomy and Comparative Pathology, Faculty of Veterinary Medicine, ‘International Excellence Agrifood Campus, CeiA3’, Córdoba, Spain; 4 Department of Animal Health, Faculty of Veterinary Medicine, Complutense University, Madrid, Spain; 5 VISAVET Health Surveillance Centre, Faculty of Veterinary Medicine, Complutense University, Madrid, Spain; Fundació Institut d’Investigació en Ciències de la Salut Germans Trias i Pujol, Universitat Autònoma de Barcelona, SPAIN

## Abstract

Tuberculosis-like lesions (TBL) in pigs have been associated with microorganisms other than mycobacteria. In this work a histopathological and microbiological evaluation of TBL in pigs is shown. A total of 352 samples belonging to 171 pigs totally condemned at slaughterhouse due to generalized TBL were sampled and selected for analysis. Pyogranulomatous (56.2%) and granulomatous lesions (20.2%) were observed in all analysed organs. Most of the granulomas observed in both lymph nodes and lungs belonged to more advanced stages of development (stages III and IV) whereas in the liver and the spleen most of lesions belonged to intermediate stages (stages II and III). Different microorganisms were simultaneously detected from TBL in the 42.7% of the animals. *Mycobacterium tuberculosis* complex (MTC) (38%), coryneform bacteria (40.3%) and streptococci (28.1%) were the main groups of microorganisms detected after bacteriological analysis, with *Trueperella pyogenes* and *Streptococcus suis* as the most frequently isolated species. Mycobacteria belonging to MTC were the most frequently detected pathogens in granulomatous and pyogranulomatous lesions in submandibular lymph nodes (32.7%) and coryneform bacteria were the microorganisms more frequently isolated from lungs (25.9%) and spleen samples (37.2%). These results may provide new insights into the pathogenesis and diagnosis of this pathology. The importance of coryneform bacteria and streptococci in such processes must be evaluated in future studies.

## Introduction

Tuberculosis-like lesions (TBL) can be an important cause of condemnation in swine at abattoir inspection representing significant important economic losses [1]. In pigs these lesions are described as necrotic-calcified, proliferative or purulent gross lesions compatible with tuberculosis (TB) [2, 3]. Although TBL in pigs are frequently limited to head lymph nodes, different body locations such as other lymph nodes and thoracic or abdominal organs can be also affected [3, 4].

Granulomatous and pyogranulomatous lesions can be identified in TBL according to the cellular components [5]. Granulomas, as the main lesions associated with TB, have been widely classified within different stages of development that may help in the interpretation of disease progression [4, 6, 7, 8]. More advanced stages of granulomas have been associated with primary sites of infection [3, 4], but also with a lower bacterial load [3, 6].

*Mycobacterium avium* complex (MAC), *Mycobacterium tuberculosis* complex (MTC) and *Rhodococcus equi* have been reported as the species most frequently associated with TBL, and these infections typically result in indistinguishable gross lesions in pigs [5, 9, 10, 11, 12, 13]. Other genera such as *Corynebacterium* spp., *Streptococcus* spp. or *Staphylococcus* spp., have also been isolated in caseous lymphadenitis in pigs, highlighting the potential diversity of pathogens that might be associated with TBL in this species [1, 14, 15]. This diversity of microorganisms together with the zoonotic nature of several of them, are factors that should be considered by public health authorities [1].

Detailed studies evaluating the relative importance of microorganisms other than *Mycobacterium* spp. identified from TBL in pigs are scarce [1, 10]. In this work a histopathological and microbiological evaluation of TBL in pigs is shown. Results of this study can help to better understand the interaction among microorganisms in pigs affected by TBL to improve the knowledge on the pathogenesis and diagnosis of this pathology.

## Material and Methods

### Ethics statement

This study did not involve purposeful killing of animals. Samples were collected from pigs after routine slaughter and meat inspection procedures. No ethical approval was deemed necessary.

### Study design and sampling

**Fig 1:**
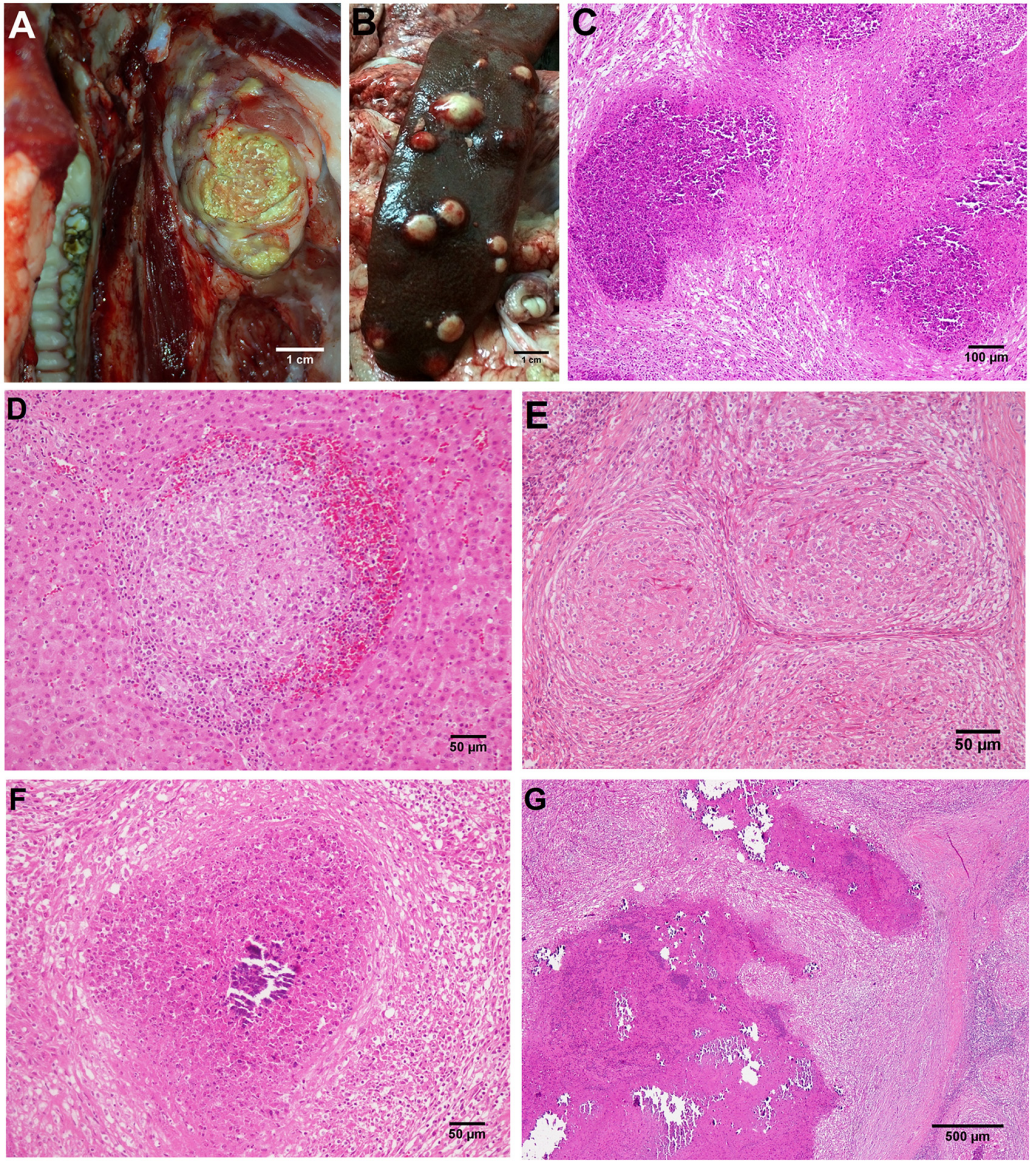
A-G. **A)** TBL in the submandibular lymph node of an affected pig. Bar, 1cm. B) TBL in the spleen of an affected pig. Bar, 1cm. C) Microscopic image of a TBL lesions in the lymph node of an affected animal showing a profuse infiltrate of degenerated neutrophils. HE. Bar, 200μm. D) Clustered epithelioid macrophages surrounded by lymphocytes and erythrocytes in a stage I granuloma in the liver. HE. Bar, 50μm. E) Coalescent stage II granulomas in the lymph node of a pig showing epithelioid macrophages completely enclosed by a thin capsule, with peripheral infiltration of scattered lymphocytes. HE. Bar, 100μm. F) Stage III granuloma with a central necrotic core, partially mineralized, surrounded by a dense connective tissue capsule infiltrated by lymphocytes and scattered neutrophils. HE. Bar, 100μm. G) Thickly encapsulated, large, irregular, multicentric granulomas with prominent caseous necrosis and multifocal islands of mineralization (stage IV granulomas). HE. Bar, 500μm.

A total of 171 pigs where the carcasses were totally condemned due to the identification of generalized disease according to the European regulation for meat inspection (Regulation 2004/854/EC) were sampled at two different slaughterhouses between January 2011 and June 2014. All animals passed antemortem clinical inspection were apparently healthy free-range pigs over 14 month-old raised in extensive systems from 56 farms located in South West Iberian Peninsula (Andalusia and Extremadura regions in Spain). After meat inspection procedures selected organs affected by TBL were sampled according to previous reports [2,3] including submandibular lymph nodes, lungs, liver and spleen to evaluate disseminated lesions [3,4,9] (Fig 1A and 1B). From these animals, a total of 352 samples were removed at the slaughterhouse and transported to the laboratory for analysis. To avoid cross contamination, different sets of sterile instruments and vials were used to collect and transport samples from each animal. Whenever possible, one well-defined lesion was selected in each organ which was divided into two portions: one portion was subjected to histopathological analysis and the other was immediately submitted to bacterial culture and frozen at -20°C to perform qPCR assays [12]. However, when small-sized disseminated lesions were observed, lesions that were similar in appearance and concentrated in one locality were selected and submitted to each analysis.

### Histopathological analysis

Submandibular lymph nodes, lungs, liver and spleen tissue samples were fixed in 10% neutral buffered formalin and 4 μm sections were stained with haematoxylin and eosin for histopathological examination and by the Ziehl-Neelsen (ZN) method to detect acid-fast bacilli (AFB) by light microscopy [2, 8]. Each sample was classified according to the identification of specific structures, namely, epithelioid cell, multinucleated giant (MNG) cell, lymphocyte and/or neutrophil infiltration, connective tissue capsule formation, antigen-antibody deposits, necrosis and mineralization. The presence of granulomas with epithelioid cells and MNG cells in the absence of foreign bodies or fungal structures was considered compatible with a diagnosis of TB. Granulomas were classified into four stages (I–IV) based on the pathological characterization of TB granulomas previously described [7, 8] (Fig 1D–1G). Lesions characterized by a necrotic core with an abundant neutrophil infiltration surrounded by epithelioid cells and a rim of connective tissue with infiltrate of mononuclear cells were described as pyogranulomas (Fig 1C).

### TB diagnosis

The presence of MTC and MAC in the lesions was tested using a duplex real time PCR (qPCR) previously validated by our research group [16]. Fat and connective tissue were removed from affected organs and lesions were subsequently minced into small pieces with sterile scissors. For every sample, up to 2 g of tissue were homogenized in a stomacher with 10 mL of sterile distilled water for a duration of 2 min. The obtained solution was centrifuged for 10 min at 1,400 *g* resulting in a pellet for each sample. Genomic DNA was extracted from 25 mg of tissue homogenate using NucleoSpin® Tissue DNA isolation kit (Macherey-Nagel GmbH, Düren, Germany) according to the manufacturer’s instructions. DNA yields and quality were determined using a NanoDrop 3300 spectrophotometer (Thermo scientific).

All reactions were run in duplicate in a Agilent Technologies Mx3000P thermocycler under the following conditions: initial denaturation at 95°C for 10 min, 40 cycles of amplification consisting of denaturation at 95°C for 30 sec, primer annealing at 65°C for 30 sec, and extension at 72°C for 30 sec. To check the specificity of the amplified products, DNA from *M*. *bovis* and *M*. *avium* isolates and non template controls were included in each assay and used as positive and negative controls, respectively.

ZN staining was performed in tissue samples negative to qPCR and examined for AFB as described by Santos et al. [2].

### Bacterial isolation

A swab from each sample submitted to bacterial culture was plated on Blood Agar Base and Columbia Blood Agar Base with nalidixic acid and colistin sulfate (Oxoid ltd., Hampshire, UK), supplemented with 5% sterile defibrinated sheep blood and incubated both in aerobic and microaerophilic (5% CO_2_) conditions at 37°C for 48 h. One representative colony of the most abundant morphologically distinct colonies were selected, subcultured and grown in the same conditions for further biochemical identification. Gram staining, bacterial morphology and production of catalase and cytochrome oxidase were performed as preliminary identification tests according to standard procedures [17]. Further biochemical identification was performed using commercial identification galleries (API^®^Coryne, API^®^20Strep, API^®^20E and API^®^20NE, bioMérieux, Marcy-l’Etoile, France) according to manufacturer’s instructions. Isolates were identified as a particular species only if identification scores in the multi-substrate identification systems were excellent, very good or good (99.9–99.0% ID); otherwise, identification was made only at the genus level (spp.). Latex agglutination test for the identification of streptococcal groups (Streptococcal grouping kit, Oxoid ltd, Hampshire, UK), and Christie Atkins Munch-Petersen test (CAMP test) were used for identification if necessary according to previous reports [18, 19, 20]. Pure cultures of each isolate were stored at -70°C.

### 16S rRNA gene sequencing

Coryneform bacteria isolates (Gram-positive, catalase variable and oxidase negative irregularly shaped rods) were identified applying 16S rRNA gene sequencing due to the limited capacity of biochemical methods to discriminate between species [21]. The 16S rRNA gene of each isolate was amplified by PCR and further sequenced to determine genotypic identity [22]. The determined sequences consisted of about 1,400 nucleotides and were compared with the sequences of other Gram-positive species available in the GenBank database, by using the FASTA program (http://www.ebi.ac.uk/fasta33).

## Results

### Histopathological analysis

A total of 352 samples from 171 slaughtered pigs with TBL were evaluated. Pyogranuloma was the lesion most frequently detected in all the examined organs (198/352; 56.2%) (Table 1) in approximately 60% of the animals (104/171; 60.8%).

**Table 1:** Type of lesions identified from samples.

	Total	SLN^a^	Lungs	Liver	Spleen
	N° (%)	N° (%)	N° (%)	N° (%)	N° (%)
**Pyogranuloma**	198 (56.2)	94 (60.3)	42 (49.4)	33 (48.5)	29 (67.4)
**Granuloma**	71 (20.2)	38 (24.4)	14 (16.5)	14 (20.6)	5 (11.6)
**Necrosis or calcification**	12 (3.4)	8 (5.1)	0 (0)	4 (5.9)	0 (0)
**Other lesions**	33 (9.4)	2 (1.3)	23 (27.1)	7 (10.3)	1 (2.3)
**No lesions**	38 (10.8)	14 (8.9)	6 (7.1)	10 (14.7)	8 (18.6)
**Total**	352 (100)	156 (100)	85 (100)	68 (100)	43 (100)

^a^SLN: submandibular lymph nodes.

Granulomatous lesions were observed in 71/352 (20.2%) of samples, being described in 38/171 of animals (22.2%). The presence of concomitant pyogranulomatous and granulomatous lesions in different organs was observed in 14 out of 171 animals (8.2%). Necrotic foci or lesions showing intense mineralization and fibrosis, with absence of epithelioid cells or MNGCs (13/352; 3.7%) were separately considered as these lesions could not clearly be classified as either pyogranulomas or granulomas. Other lesions such as interstitial pneumonia, catarrhal-purulent bronchitis, periportal fibrosis, periesplenitis and interstitial and multifocal hepatitis were also detected in the absence of granulomatous or pyogranulomatous lesions (71/352, 20.2% samples; and 1/171, 0.6% animals). Finally, in a reduced number of cases, lesions could not be detected (38/352, 10.8% samples; and 9/171, 5.3% animals).

Granulomas were mostly observed in samples from submandibular lymph nodes and to a lesser extent in liver, lungs and spleen (Table 1). Regarding the stage of granulomas, 31% of granulomas belonged to the initial stages (I and II), whereas 69% of the granulomas were included within the stages III and IV. This pattern was confirmed for lymph node and lung samples, whereas in the spleen and the liver most of the granulomas belonged to the stages II and III (Table 2).

**Table 2:** Distribution of granulomas per examined organ and stage of development.

	Total	SLN^a^	Lung	Liver	Spleen
	N° (%)	N° (%)	N° (%)	N° (%)	N° (%)
**Stage I**	2 (2.8)	0 (0)	1 (7.7)	1 (7.1)	0 (0)
**Stage II**	20 (28.2)	10 (25.6)	3 (23.1)	5 (35.7)	2 (40)
**Stage III**	27 (38)	16 (41)	3 (23.1)	6 (42.9)	2 (40)
**Stage IV**	22 (31)	13 (33.3)	6 (46.1)	2 (14.3)	1 (20)
**Total**	71 (100)	39 (100)	13 (100)	14 (100)	5 (100)

^a^SLN: submandibular lymph nodes.

### TB diagnosis

The genome of MTC was amplified on samples from 65 animals (65/171 animals; 38%). In 44 out of these 65 animals generalized TBL affecting submandibular lymph nodes and other organs (lungs, liver or spleen) were detected. In 25 out of these 44 animals (56.8%) MTC was detected only in submandibular lymph nodes; in 6/44 (13.6%) mycobacteria were detected in submandibular lymph nodes and lungs, liver and/or spleen; whereas in 13/44 animals (29.5%) mycobacteria were detected only in lungs, liver or spleen (data not shown). MAC was detected only in one case associated with pyogranulomatous lesions in a liver.

AFB were recorded in 15/269 (5.6%) of qPCR negative samples and in 9/105 (8.6%) of qPCR negative animals by ZN staining.

### Bacterial isolation

A total of 235 isolates were obtained after bacteriological culture (Table 3). Due to the high number of bacterial species detected in low percentages the analysis was focused on coryneform bacteria and streptococci as the main groups of microorganisms detected in this study besides MTC. Coryneform bacteria were identified in 100 out of 235 isolates (42.5%) and were recovered from 69 animals (40.3%). Most coryneform microorganisms were identified as *Trueperella pyogenes* (formerly *Arcanobacterium pyogenes*) (72%), which was isolated from a significant number (69.6%) of examined animals (Table 3). Streptococci were also identified in a notable number of isolates (65/235; 27.7%) and animals (48/171; 28.1%). *Streptococcus suis* was the species most frequently identified within this group (40% of isolates and 47.9% of animals in which streptococci were isolated) followed by *Streptococcus porcinus* and *Streptococcus dysgalactiae* spp. *equisimilis* (Table 3).

**Table 3:** Microorganisms isolated from lesions.

	Isolates		Positive animals	
	N°	%	N°	%
**Coryneform bacteria**	**100**	**42.5**	**69**	**40.3**
*Trueperella pyogenes*	72/100	72	48/69	69.6
*Corynebacterium suicordis*	12/100	12	10/69	14.5
*Rhodococcus equi*	3/100	3	3/69	4.3
*Corynebacterium xerosis*	4/100	4	2/69	2.9
*Corynebacterium* spp.	2/100	2	1/69	1.4
*Corynebacterium ulcerans*	1/100	1	1/69	1.4
*Corynebacterium urealyticum*	1/100	1	1/69	1.4
Other coryneform bacteria^a^	5/100	5	5/69	7.2
**Streptococci**	**65**	**27.7**	**48**	**28.1**
*Streptococcus suis*	26/65	40	23/48	47.9
*Streptococcus porcinus*	12/65	18.5	7/48	14.6
*Streptococcus dysgalactiae* spp. *equisimilis*	6/65	9.2	5/48	10.4
*Streptococcus equi* spp. *zooepidemicus*	5/65	7.7	4/48	8.3
*Streptococcus agalactiae*	4/65	6.1	4/48	8.3
*Streptococcus alactolyticus*	4/65	6.1	2/48	4.2
*Streptococcus uberis*	2/65	3.1	2/48	4.2
Other streptococci^b^	6/65	9.2	6/48	12.5
***Enterococcus* spp.** ^c^	19	8.1	14	8.2
***Carnobacterium* spp.** ^d^	17	7.2	14	8.2
***Aerococcus* spp.** ^e^	13	5.5	11	6.4
***Staphylococcus* spp.** ^f^	7	3	7	4.1
***Pasteurella multocida***	4	1.7	4	2.3
**Others** ^**g**^	10	4.2	7	4.1
**Total**	235	100	171	100

^**a**^ *Rhodococcus boritolerans*, *Dietzia timorensis*, *Pseudoclavibacter* spp, *Brevibacterium* spp and *Actinomyces masicol* (1 isolate/each)

^b^ *Streptococcus* spp. (3 isolates), *S*. *mitis*, *S*. *rattus* and *S*. *bovis* (1 isolate/each)

^c^ *E*. *faecium* (8 isolates) *E*. *durans* (3 isolates), *E*. *faecalis* (6 isolates), *E*. *gallinarum* and *E*. *avium* (1 isolate/each)

^d^ *C*. *maltaromaticum* (16 isolates) and *C*. *divergens* (1 isolate)

^e^ *A*. *urinae* (7 isolates), *A*. *viridans* (4 isolates) and *A nurinaequi* (2 isolates)

^f^ *S*. *sciuri*, *S*. *xylosus* (2 isolates/each), *Staphylococcus* spp., *S*. *aureus* and *S*. *haemolyticus* (1 isolate/each)

^**g**^ *Leuconostoc* spp. (4 isolates), *Escherichia coli* (2 isolates) *Mezorhizobium* spp., *Halospirulina* spp., *Glanulicatella* spp. and *Lactococcus lactis* (1 isolate/each)

### Organic distribution of identified microorganisms

The frequency of detection of microorganisms in TBL from the examined organs is shown in Table 4. MTC, coryneform bacteria and streptococci were detected in all analysed organs. MTC was more frequently detected in submandibular lymph nodes (32.7%), whereas coryneform bacteria were more frequently isolated from lungs (25.9%) and spleen (37.2%). *T*. *pyogenes* was the species identified in over 60% of cases associated with coryneform bacteria in all examined organs (data not shown). Streptococci were equally isolated from lesions of all examined organs.

**Table 4:** Frequency of detection of microorganisms from TBL within the examined organs.

	Total	SLN^a^	Lungs	Liver	Spleen
	N° (%)	N° (%)	N° (%)	N° (%)	N° (%)
MTC	82 (23.3)	51 (32.7)	12 (14.1)	14 (20.6)	5 (11.6)
Coryneform bacteria	98 (27.8)	46 (29.5)	22 (25.9)	14 (20.6)	16 (37.2)
Streptococci	62 (17.6)	30 (19.2)	16 (18.8)	10 (14.7)	6 (13.9)
Others	63 (17.9)	28 (17.9)	15 (17.6)	13 (19.1)	7 (16.3)

^a^SLN: submandibular lymph nodes.

### Microorganisms and type of lesions at individuals

The bacteria identified from different type of lesions are summarized in Table 5. Twenty-three animals (13.4%) yielded negative results both by microbiological and qPCR studies despite having presented microscopic lesions. In 75 (43.9%) of the pigs, a single microorganism was identified, whereas in 73 (42.7%) of the animals two or more microorganisms were detected. The isolation of coryneform bacteria from MTC positive pigs was frequent (10.5%), with *T*. *pyogenes* being identified in 77.8% (14/18) of these cases. Moreover, different species of coryneform bacteria were isolated from MTC negative pigs (16.9%), with *T*. *pyogenes* as the main species identified (17/29; 58.6%).

**Table 5:** Frequency of detected microorganisms and type of lesions identified at individual level.

	Total		Granuloma		Pyogranuloma		Concomitant lesions^a^		Other lesions^b^	
	N°	%	N°	%	N°	%	N°	%	N°	%
**MTC positive animals**	**65**	**38**	**19**	**50**	**34**	**32.7**	**7**	**50**	**5**	**33.3**
Coryneforms	18	10.5	2	5.2	16	15.4	0	0	0	0
Streptococci	6	3.5	1	2.6	2	1.9	0	0	3	20
Others	9	5.3	3	7.9	4	3.8	1	7.1	1	6.7
Coryneforms + streptococci	10	5.8	4	10.5	4	3.8	2	14.3	0	0
Coryneforms + others	4	2.3	1	2.6	2	1.9	1	7.1	0	0
Streptococci+ others	3	1.7	2	5.3	1	1	0	0	0	0
No isolation	15	8.8	6	15.8	5	4.8	3	21.4	1	6.7
**MTC negative animals**	**106**	**62**	**19**	**50**	**70**	**67.3**	**7**	**50**	**10**	**66.7**
Coryneforms	29	16.9	1	2.6	24	23.1	1	7.1	3	20
Streptococci	17	9.9	1	2.6	10	9.6	2	14.3	4	26.7
Others	22	12.9	6	15.8	12	11.5	1	7.1	3	20
Coryneforms + streptococci	4	2.3	1	2.6	3	2.9	0	0	0	0
Coryneforms +streptococci + others	1	0.6	0	0	1	1	0	0	0	0
Coryneforms + others	3	1.7	2	5.3	0	0	1	7.1	0	0
Streptococci + others	7	4.1	2	5.3	5	4.8	0	0	0	0
No isolation	23	13.4	6	15.8	15	14.4	2	14.3	0	0
**Total**	171	100	38	100	104	100	14	100	15	100

^a^Granulomatous and pyogranulomatous lesions detected in the same animal

^b^Necrotic or calcified foci (5/15) and other lesions or no lesions (10/15)

Mycobacteria belonging to MTC were identified in 19/38 (50%) of pigs in which granuloma was the unique detected lesion (Table 5). Bacteria other than mycobacteria were also detected from granulomatous lesions in 13/38 of animals (34.2%), whereas no microorganisms were identified in 6/38 (15.8%). However, when pyogranuloma was considered as a sole lesion, most animals were negative to MTC (70/104; 67.3%) (Table 5). Coryneform bacteria (50/104; 48.1% animals) and less frequently streptococci (26/104; 25% animals) were the main microbial agents isolated from these lesions.

## Discussion

Tuberculosis-like lesions include a wide range of lesions grossly compatible with TB [2, 3]. However, previous studies have shown that pathogens other than mycobacteria may cause indistinguishable lesions in pigs [10]. The involvement of different pathogens in the development of these lesions needs to be evaluated to assess an accurate diagnosis of TBL in pigs and to establish effective control measures against this disease [1].

In this study, pyogranuloma was the predominant lesional pattern (104/171 animals; 60.8%) with granuloma being detected only in 38 out of 171 animals (22.2%). The high number of pyogranulomatous lesions detected in the present study (198/352 samples; 56.2%) suggests the importance of pyogenic bacteria in the etiology of TBL in pigs. In fact, a wide spectrum of bacteria belonging to twenty different genera was detected. MTC was detected in 65 (38%) of the animals, together with an important participation of coryneform bacteria and streptococci (40.35% and 28.1% positive animals respectively). These findings reinforce the multi-etiological nature of TBL.

The detection of multiple microbial agents was frequent (42.7% of analysed animals) highlighting the importance of performing a thorough microbiological examination of TBL for disease surveillance [1]. MTC, coryneform bacteria (including *T*. *pyogenes*, *Corynebacterium* spp., and related genera) and streptococci were the pathogens more frequently detected from TBL. Other microorganisms also identified but with a lower frequency (*Staphylococcus* spp., *Pasteurella multocida*, *Enterococcus* spp., *Carnobacterium* spp., *Aerococcus* spp.) can be isolated from the environment, faeces, skin and mucous membranes of pigs [23], but their importance in this process is unknown. Therefore, our analysis was focused on the most representative groups of pathogens identified in the study.

MTC and MAC may play different roles in TBL according to the prevalence of bovine TB. In this sense, *M*. *avium* is the main mycobacteria recovered from TBL in officially bovine TB free countries [24] as well as in fattening pigs reared in intensive systems [25]. However, in countries where TB is still prevalent in cattle and wildlife MTC is frequently detected from TBL in free-range pigs [2, 9, 13]. Pigs of this study were bred in a free-ranged system sharing natural resources with other wild and domestic animals in a geographical area in which a high prevalence of TB infected wild boars has been described [26]. Contact and cross infection between these populations may occur as has been reported in other areas of Spain [9]. Accordingly, MTC was predominately detected from TBL in our study.

The frequency of isolation of *Rhodococcus equi* in our study (4/171, 2.3% animals) was much lower than previously reported in pigs, negative to mycobacteria, and reared in intensive farms in the Netherlands [10]. Despite further studies being deemed necessary to elucidate these differences, several factors such as breed susceptibility, herd management and the ecology of the bacteria may play a role. Corynebacteria other than *Rhodococcus equi* have been sporadically related with severe caseous lymphadenitis in pigs, including *Corynebacterium ulcerans* and *Corynebacterium pseudotuberculosis* [14, 15]. In the present study, *Corynebacterium suicordis* was the main species isolated within this genus, but its relative importance was low. *T*. *pyogenes* was the predominant species within this group. This pathogen is involved in miscellaneous pyogenic infections in pigs and ruminants, including metritis, udder lesions, abscesses, pneumonia, arthritis, endocarditis, lymphadenitis and osteomyelitis [27, 28, 29]. Although *T*. *pyogenes* has previously been associated with caseous lymphadenitis in pigs [1], its relative importance was low in comparison with our study.

Similarly to Lara et al. [1] streptococci were detected in a high percentage of animals. Interestingly, *S*. *suis* was the species most frequently identified within this group. This microorganism has been associated with a wide variety of diseases in pigs such as meningitis, arthritis, bronchopneumonia, endocarditis, poliserositis and septicaemia and has been considered as an emerging zoonotic agent in humans secondary to exposition to pigs and pork products [30]. The other two streptococcal species more frequently isolated, *Streptococcus porcinus* and *Streptococcus dysgalactiae* spp. *equisimilis*, are also frequently isolated from pigs with suppurative infections [27, 31].

Negative bacteriological results were observed in accordance with previous reports [1, 2, 32]. These results may be attributed to false negative results of bacteriology or animals with advanced lesions in which viable microorganisms could not be obtained. In this sense, the 21.7% (5/23) of animals that showed microscopic lesions but were negative to both bacterial culture and qPCR, showed AFB on histopathological examination suggesting possible mycobacterial involvement in several of them.

Coryneform bacteria isolation from MTC negative TBL was the pattern most frequently observed from animals affected by pyogranulomatous lesions (24/104; 23.1%). These results support the idea that pyogenic bacteria can originate TBL in pigs without the involvement of mycobacteria. In this sense, although false negative results of the qPCR analysis should be considered, AFB were only recorded in lesions of two of these animals. Alternatively, simultaneous detection of coryneform bacteria and MTC was also frequent, suggesting a possible involvement of this microbial association in pigs affected by TBL.

Granulomas were predominately observed in submandibular lymph nodes and to a lesser extent in other body locations. The submandibular lymph node was the organ in which MTC was more frequently detected, followed by the liver, lungs and spleen. Interestingly, MTC was identified in a similar rate from both liver and lung samples, with most of the granulomatous lesions belonging to stages III and IV. These results support the theory that both the respiratory and digestive routes of infection play an important role in pigs, as previously suggested [4, 9].

Despite several authors have suggested that generalized TB in swine is frequent [3, 33], others have reported a restriction of TBL to head lymph nodes or less frequently to the respiratory tract [2, 9, 34]. Our results are in agreement with this latter statement, since MTC was detected only in submandibular lymph nodes in more than half of the animals. Interestingly, pyogenic bacteria, including *T*. *pyogenes*, *Streptococcus* spp., and *Corynebacterium* spp., were isolated from TBL observed in other organs from these MTC-positive animals (data not shown). These findings should be taken into account to avoid misdiagnosis of generalized TB based on gross inspection and to carry out further studies to determine the true role of these agents, especially *T*. *pyogenes* in pyogranulomatous lesions in pigs

## Conclusions

The results of this study show that a wide spectrum of microorganisms different to mycobacteria can be isolated from TBL in pigs, with coryneform bacteria and streptococci as the microorganisms most frequently detected besides MTC. The high frequency of detection of *T*. *pyogenes* in pyogranulomatous lesions is also shown. These results should be considered to prevent misdiagnosis of TB based on gross lesions and to establish specific control measures against these pathogens in pigs.
